# Ultrasound-Assisted Extraction of Squalene and 2-Acetyl-1-Pyrroline from Pandan Leaf: The Effects of Drying Methods and Extraction Conditions

**DOI:** 10.3390/foods13244010

**Published:** 2024-12-11

**Authors:** Yanfei Cheng, Tao Fei, Yuyi Liu, Shuai Chen, Zexin Wang, Yiran Han, Lu Wang, Congfa Li

**Affiliations:** 1 School of Food Science and Engineering, Hainan University, Haikou 570228, China; chengyanfei1620@163.com (Y.C.); feitao_fse@hainanu.edu.cn (T.F.); 22220951350082@hainanu.edu.cn (Y.L.); 17674726015@163.com (S.C.); 18313570568@163.com (Z.W.); hanyiran0306@163.com (Y.H.); lwang@hainanu.edu.cn (L.W.); 2 Key Laboratory of Food Nutrition and Functional Food of Hainan Province, Haikou 570228, China; 3 Key Laboratory of Tropical Agricultural Products Processing Technology of Haikou, Haikou 570228, China

**Keywords:** Pandan leaf, ultrasound-assisted extraction, extraction solvents, squalene, antioxidant, hypoglycemic

## Abstract

Pandan, a tropical crop, is rich in squalene (SQ), known for its antioxidant and hypoglycemic properties, and 2-acetyl-1-pyrroline (2-AP), which imparts a characteristic aroma. This study focuses on the extraction of the two bioactive compounds from Pandan leaves and investigates the effects of drying methods, extraction solvents, and conditions on the yield of SQ and 2-AP. Results show that hot air-dried Pandan leaves when extracted using the binary solvent system of ethanol and n-hexane (EH), yield higher SQ content while maintaining an adequate content of 2-AP. To further optimize the extraction process, a single-factor experiment was followed by optimization using Box–Behnken design (BBD) and response surface methodology (RSM). The optimal extraction conditions were determined as follows: ultrasound time of 60 min, a temperature of 50 °C, power of 300 W, and a solid-to-liquid ratio of 1:5 g/mL. Under these conditions, an SQ yield of 1229.98 ± 13.09 μg/DW 1 g Pandan leaves and a 2-AP yield of 80.72 ± 0.88 μg/DW 1 g Pandan leaves were achieved, representing increases of 3.30% and 9.82% compared to pre-optimization values. Additionally, the antioxidant activities of EH extracts were evaluated through various in vitro assays. The extracts demonstrated significant DPPH and ABTS free radical scavenging activity (12.46 μmol TE/g DW and 22.14 μmol TE/g DW, respectively), along with ferric and cupric ion reducing power (10.629 μmol TE/g DW and 14.275 μmol TE/g DW, respectively). The extracts also exhibited notable inhibitory effects on α-amylase and α-glucosidase. The findings suggest that these extracts are a promising natural source of antioxidants with potential applications in health and nutrition.

## 1. Introduction

Pandan (*Pandanus amarylifolius Roxb*.) is a tropical plant widely distributed in the moist tropics of Africa, Asia, and the Pacific. It is the only Pandanus species known to contain fragrant compounds [1]. The plant is characterized by its strap-shaped leaves, which taper towards the tip. Pandan leaves are highly valued for their medicinal properties, owing to the abundance of bioactive compounds of polyphenols and flavonoids (catechin, gallic acid, and kaempferol, etc.), alkaloids (pandamarilactonine A and B, etc.), and terpenoids such as Squalene (SQ) [2,3]. SQ is a natural polyunsaturated triterpene mainly sourced from shark liver oil and is considered an effective chemopreventive and chemotherapeutic agent [4]. However, the increasing demand for SQ coupled with regulatory constraints and concerns about marine pollution, has driven interest in plant-derived alternatives [5]. Among various sources, like Soybean oil and peanuts with lower SQ contents [6], Pandan has emerged as a promising candidate for sustainable plant-sourced SQ production. In addition to SQ, Pandan extracts consist of various volatile compounds like 2-acetyl-1-pyrroline (2-AP), ethyl formate, 3-hexanone, 2-hexanone, 4-methylpentanol, trans-2-heptenal, 3-hexanol, and β-damascenone, etc. [7]. Among these, 2-AP is responsible for the distinct aroma of Pandan leaves. This compound is commonly used as a natural flavoring agent or food ingredient in a variety of foods including sweets, pastries, cakes, etc. [8,9]. Given the dual value of SQ and 2-AP, developing an efficient extraction method is crucial for addressing the growing demand for plant-sourced SQ while enhancing the economic potential of Pandan.

The extraction process plays a pivotal role in isolating bioactive compounds from natural plant materials [10]. The selection of extraction methods is essential for achieving the best efficiency. Traditional extraction methods, such as leaching, percolation, and reflux, are often time-consuming and yield low extraction efficiency. To overcome these limitations, emerging extraction technologies, such as ultrasound-assisted (UA) extraction, microwave-assisted extraction, accelerated solvent extraction and pressurized liquid extraction, enzyme-assisted extraction, supercritical fluid extraction, and deep eutectic solvents are superior to conventional techniques due to their high extraction efficiency, high selectivity, short extraction times, environmental friendliness and safety [11]. Among these, UA which improves the yields of various phytochemicals and reduces energy requirements, space requirements, extraction time, maintenance cost, and carbon dioxide emissions, [12,13], has been widely used to extract bioactive compounds from natural products [14,15]. Ultrasound cavitation can inactivate enzymes, disrupt cell walls, and lead to increased contact between the cell and the solvent contents, thereby promoting the rapid release of bioactive compounds [16,17]. In addition to the extraction method, other factors such as drying methods and extraction solvents may also significantly affect the yield of targeted compounds [18]. However, limited research has focused on optimizing the UA extraction of SQ and 2-AP from Pandan leaves, particularly in relation to these variables.

This study aims to optimize the simultaneous extraction of SQ and 2-AP from Pandan leaves. First, the effects of various drying methods and extraction solvents on the yield of SQ and 2-AP are evaluated using UA. Next, the influence of key parameters, including ultrasound time, temperature, power, and the solid-to-liquid ratio, is investigated. Response surface methodology (RSM) is then employed to optimize these conditions for maximum yield. Finally, the bioactivities of the extracts are assessed through in vitro antioxidant assays (DPPH, ABTS, FRAP, and CUPRAC) and enzyme inhibition assays targeting *α*-glucosidase and *α*-amylase, providing data and theoretical support for the potential use of the extracts as a source of antioxidant and hypoglycemic agent in future food and pharmaceuticals industrial applications.

## 2. Materials and Methods

### 2.1. Materials Preparation and Solvents

Pandan leaves were harvested in July 2023 from the farm in Qionghai city, Hainan, China. The hand-picked Pandan leaves were placed in a foam box with ice packs and transported to the laboratory of the School of Food Science and Engineering, Hainan University, China. SQ standard was purchased from Sigma Aldrich Chemical Co., Ltd. (Shanghai, China). 2-AP standard was purchased from Toronto Research Chemicals (TRC, North York, ON, Canada). Acarbose (≥99.7%) and p-nitrophenyl-*α*-D-glucopyranoside (PNPG, ≥99%) were obtained from Sigma-Aldrich (Shanghai, China). All other chemical reagents were analytical grade.

Based on our previous experiments for the determination of the moisture content (82–85%) in Pandan leaves, the fresh Pandan leaves were dried using the hot air drying oven (WGL-125B, Tianjin Taiwo Instrument Co., Ltd., Tianjin, China) at 50 °C for 36 h and a freeze dryer (FD-1A-50, Shanghai Zuo Le Instrument Co., Ltd., Shanghai, China) for 48 h until a residue moisture content of 6–10%. The dried Pandan leaves were powdered using a lab grinder, sieved through the 60-mesh sieve, and then stored for the extraction of SQ and 2-AP.

### 2.2. Ultrasound-Assisted Extraction of SQ and 2-AP Using Different Solvents

Based on the polarity of SQ and 2-AP, the like-dissolves-like rule and preliminarily experimental studies, the following extraction solvents were used to extract SQ and 2-AP from Pandan leaves using UA: the individual system of ethanol (EtOH), isopropanol (IPA) and n-hexane (Hex), the binary system of ethanol: isopropanol (EI), ethanol: n-hexane (EH), isopropanol: n-hexane (IH) with a volume ratio of 1:1 (*v*/*v*). According to the extraction conditions reported by Chanioti and Tzia [19] with some modifications, the UA was performed under the following conditions: ultrasound time, 60 min, ultrasound temperature, 50 °C, ultrasound power, 360 W, and solid-to-liquid ratio, 1:10 g/mL (GL1222, Shenzhen Guanbo Technology Industry Co., Ltd., Shenzhen, China). After the extraction, the solution was centrifuged (LGJ-10NS, Beijing, China). With the selection of suitable solvents and drying methods from the above six different extraction solvents and two drying methods, ultrasonication conditions were then optimized. After extraction and centrifugation, the supernatant was filtered before storage at −20 °C for further analysis.

### 2.3. Scanning Electron Microscope (SEM)

After the UA, these treated Pandan powders were collected, dried, and then analyzed using SEM to investigate the influence of various drying methods (hot air and freeze drying) on the structure of Pandan powder before and after UA.

### 2.4. Single Factor Experiment

Optimization of UA conditions using single-factor experiments includes A (ultrasound time: 40–70 min), B (ultrasound temperature: 30–60 °C), C (ultrasound power: 300–480 W), and D (solid to liquid ratio: 1:5–1:20 g/mL). The dependent variables were the SQ content and the 2-AP content. Each experiment was conducted in triplicates.

### 2.5. Response Surface Methodology Model (RSM)

RSM was employed to figure out the interaction and the influence between the independent variables including ultrasound time (A), ultrasound temperature (B), and ultrasound power (C) on the extraction content of SQ and 2-AP (Table 1). The experiment data were designed using BBD with the software package Design-Expert 13.0. The experiments were randomized to minimize the influences of extraneous factors on unexplained variation in the observed responses. The repeatability of the method was assessed through designing five center points.

### 2.6. Characterization of Pandan Leaf Extracts

#### 2.6.1. HPLC-DAD Analysis of SQ Content

The content of SQ in the Pandan leaf extracts was determined using a Hitachi HPLC system (DAD detector, 5110, Naka City, Japan) equipped with a ZORBAX Eclipse C_18_, 5 μm, 4.6 × 250 mm column. The method employed was as described by Farjaminezhad [20] with certain modifications. All extracts were analyzed under the following conditions: mobile phase, 100% MeOH; wavelength, 210 nm; injection volume, 10 μL; flow rate, 1 mL/min.

#### 2.6.2. LC-MS/MS Analysis of 2-AP Content

The qualitative and quantitative analysis of 2-AP in 12 Pandan leaf extracts was performed by ultrahigh-performance liquid chromatography (Waters, Milford, MA, USA) coupled to (tandem) mass spectrometry (UPLC-MS/MS, SCIEX, Framingham, MA, USA). The method was adopted from Bösl [21] with some modifications. For the chromatographic conditions, a 150 mm × 2.1 mm × 1.7 μm reversed-phase column was employed on the LC process with the following parameters: the column temperature, 40 °C, a mobile phase of 0.1% formic acid in water, and acetonitrile with the ratio of 95:5 (*v*/*v*), injection volume, 1 μL. The detection was performed in positive ionization mode. 

#### 2.6.3. The Total Phenolic Content (TPC) and Total Flavonoid Content (TFC) in EH Pandan Leaf Extract 

The TPC was measured by using the Folin–Ciocalteu method [22]. A sample of 50 μL was mixed with 50 μL Folin–Ciocalteu reagent and the reaction was carried out for 10 min in the dark at room temperature. Then, 20% Na_2_CO_3_ (150 μL) and distilled water (300 μL) were added, and the sample was measured at 765 nm after 30 min. TPC was calculated through a standard curve using gallic acid (GAE) as the standard with the calibration curve of Y = 0.0048X − 0.0172, R^2^ = 0.9992. The results were presented as a milligram of gallic acid equivalents per gram of Pandan powder dry weight, (mg GAE/g DW). TFC was determined by using aluminum chloride colorimetry [22]. Na_2_NO_2_ solution (5%) of 50 μL, 50 μL 10% AlCl_3,_ and 400 μL NaOH solution (1 mmol/L) were added to the sample (100 μL). The mixture was then incubated in the dark for 30 min at room temperature. TFC was calculated using rutin as the reference with the calibration curve of Y = 0.0006X − 0.001, R^2^ = 0.9998). The results were expressed as a milligram of rutin equivalents per gram of Pandan powder dry weight, (mg RE/g DW).

#### 2.6.4. The Total Triterpenes Content (TTC) in EH Pandan Leaf Extract

The method used to determine TTC was reported by Luo [23] with modifications. Vanillin-acetic solution (5%) of 100 μL was added to a 50 μL sample and the mixture was placed at 90 °C for 5 min. Then, 200 μL perchloric acid was added and the mixture was allowed for reaction at 60 °C for 20 min. After that, absorbance was measured at 550 nm. Ursolic acid was used as a reference and the calibration curve was plotted Y = 0.0007X − 0.0116, R^2^ = 0.9974. The results were presented as a milligram of ursolic acid equivalents per gram of Pandan powder dry weight, (mg UAE/g DW).

### 2.7. Antioxidant Activity in the EH Pandan Leaf Extracts In Vitro

#### 2.7.1. DPPH and ABTS Radical Scavenging Activity

Free radical scavenging activities of the extracts from Pandan leaf using EH were presented as μmol Trolox equivalent antioxidant capacity (μmol TE/g DW), including DPPH and ABTS radical scavenging activity. Both activity experiments were performed using the method described by de Souza [24] with minor modifications. Briefly, 400 μL DPPH·and ABTS solution were mixed with 50 μL EH Pandan leaf extracts or Trolox solutions (0–150 μg/mL), respectively. Then, the reaction was carried out for 30 min in the dark at room temperature. After that, the mixtures were measured at 517 nm and 734 nm, respectively. 6-hydroxy-2,5,7,8-tetramethylchroman-2-carboxylic acid (Trolox) was severed as standard and the standard curve was plotted Y = 1.5842X − 1.6636, R^2^ = 0.9944 and Y = 0.9496X + 0.2.1301, R^2^ = 0.9983, respectively.

#### 2.7.2. Ferric Reducing Antioxidant Power (FRAP) and Cupric Reducing Antioxidant Capacity (CUPRAC)

The FRAP and CUPRAC in EH Pandan leaf extracts were examined using the method described by Wang [25] with slight modifications. The procedure for preparing FRAP solution was as follows: TPTZ solution, FeCl_3_ solution, and sodium acetate buffer were mixed at a volume ratio of 10:1:1, then preheated in a water bath (37 °C) for use in the following steps. FRAP solution of 900 μL was mixed with 30 μL EH Pandan leaf extracts or Trolox solutions (0–1000 μg/mL) to react for 30 min in the dark at room temperature. Then, the absorbance of the mixture was measured at 593 nm. For the CUPRAC assay, 30 μL EH Pandan leaf extracts or Trolox solutions were mixed with 100 μL Cu_2_SO_4_ solution, neocuproine solution, ammonium acetate buffer, and deionized water. The mixture was allowed for a reaction in the dark for 30 min at room temperature. The absorbance of the mixture was then measured at 450 nm. Trolox was used as the standard and the FRAP and CUPRAC values were both presented in μmol TE/g DW with the standard curve of Y = 0.0032X + 0.0214, R^2^ = 0.9975 and Y = 0.0007X + 0.009, R^2^ = 0.9987, respectively. 

### 2.8. Hypoglycemic Assay in the EH Pandan Leaf Extracts

The hypoglycemic assay was used to investigate the inhibitory influence of Pandan leaf extracts on *α*-amylase and *α*-glucosidase. The experiment was implemented according to the method as reported elsewhere with light modifications [26]. Acarbose was used as the positive control. Both inhibition abilities were presented as semi-inhibitory concentrations (IC_50_).

For the *α*-amylase inhibition ability assay, a 100 μL sample was mixed with 50 μL *α*-amylase and incubated in a 37 °C water bath for 5 min. Then, 50 μL amylopectin solution (1%) was added to continue the reaction for 20 min. Finally, 50 μL DNS was added to a boiling water bath for 5 min and the solution was cooled down to room temperature with ice water. After cooling, 1 mL of deionized water was added. The mixture was finally determined at the absorbance of 540 nm. The *α*-amylase inhibition ability (%) was calculated by following Equation (1):

(1) $$\alpha -\mathrm{amylase}\;(\%)\;=1-\frac{\mathrm{As}-\mathrm{Ac}}{\mathrm{As}}\times 100$$

where As and Ac are the absorbance of the sample and the absorbance of the control (blank), respectively.

For the *α*-glucosidase inhibition ability assay, after mixing 100 μL of sample with 100 μL of *α*-glucosidase, the reaction was incubated in a water bath at 37 °C for 10 min, then 100 μL of PNPG solution was added, and the incubation was continued at 37 °C for 20 min, and the reaction was finally terminated by the addition of 500 μL Na_2_CO_3_ solution (1 M). The mixture was measured at 405 nm. The *α*-glucosidase inhibition ability (%) was calculated by following Equation (2):

(2) $$\alpha -\mathrm{glucosidase}\;(\%)=\frac{\left(\mathrm{At}-\mathrm{Ac}\right)-\left(\mathrm{Bt}-\mathrm{Bc}\right)}{\mathrm{At}-\mathrm{Ac}}\times 100$$

where At, Ac, Bt, and Bc are denoted absorbance of the blank test group, blank control group, sample test group, and sample control group, respectively.

### 2.9. Data Analysis

Results obtained at least in triplicate were presented as the mean values ± SD (standard deviation). Experimental data were processed using SPSS Statistics 27.0. One-way ANOVA and *t*-test were employed to determine the significant differences between mean values. *p* < 0.05 was indicated as statistically significant.

## 3. Results and Discussion

### 3.1. Effects of Different Drying Methods and Solvents on the Content of SQ

**Figure 1:**
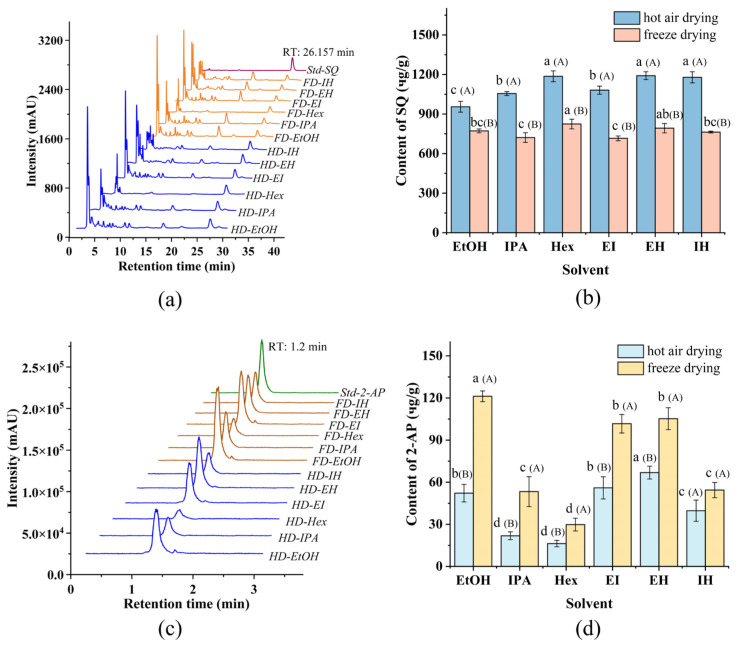
(**a**) HPLC chromatogram of Squalene (SQ) standard and Pandan leaf extracts; (**b**) SQ content: the effect of extraction solvents and drying methods; (**c**) LC-MS/MS chromatogram of 2-acetyl-1-pyrroline (2-AP) standard and Pandan leaf extracts; (**d**) 2-AP content: the effect of extraction solvents and drying methods. The lowercase letters on the line were expressed as the significance between various extraction systems. The uppercase letters (A and B) were expressed as the significance between hot air drying and freeze drying. Retention time (RT).

The HPLC chromatogram (Figure 1a) confirms the presence of SQ in all 12 extracts, with a characteristic peak at 26.157 min. However, the quantity of SQ varied significantly depending on the drying method and the type of solvent. Figure 1b presents the quantification of SQ in different solvent extracts from Pandan leaves. For hot air-dried Pandan leaves, the yield of SQ in each final extract ranged from a maximum of 1190.70 ± 29.77 μg/DW in 1 g Pandan leaves (EH extracts) to a minimum of 955.31 ± 40.82 μg/DW in 1 g Pandan leaf (EtOH extracts). Among the solvent systems, Hex extraction yielded the highest SQ content, followed by EH, with EtOH extraction yielding the least. This disparity can be attributed to the superior ability of non-polar solvents such as Hex for extracting non-polar compounds from raw materials, compared to polar solvents such as EtOH and IPA [27]. Moreover, the results of the binary solvent extraction systems demonstrated that EH extracts had the highest SQ content, surpassing the yield from the individual solvent system. A similar trend was observed for freeze-dried Pandan leaves. This can be attributed to the lower viscosity of EH. The combination of EtOH and Hex promotes favorable enthalpy and entropy interactions, reducing the Gibbs free energy of the system, thereby increasing solubility and facilitating the transport of fat-soluble compounds within the cells [6].

**Figure 2:**
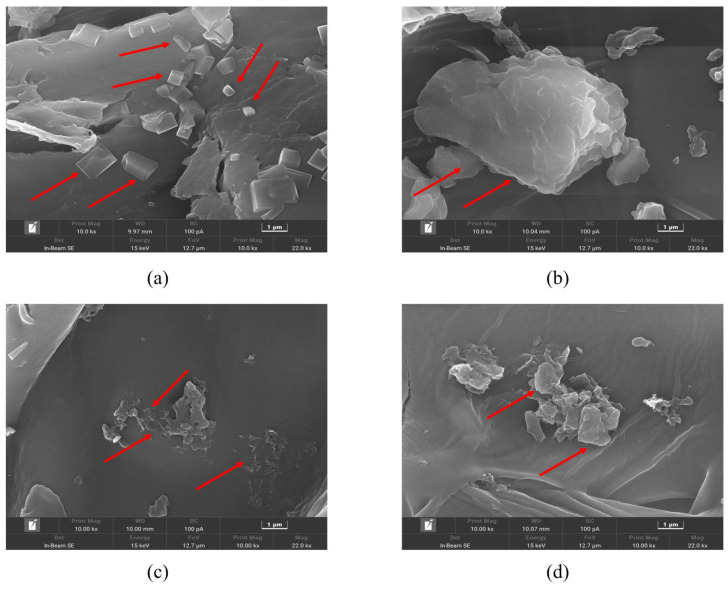
SEM images of leaf cell residues of hot air-dried Pandan powder and freeze-dried Pandan powder before and after ultrasound-assisted extraction at 10,000 magnifications disrupted. Differences can be seen where the red arrows point. (**a**) hot air-dried and (**b**) freeze-dried Pandan powder before extraction; (**c**) hot air-dried and (**d**) freeze-dried Pandan powder after extraction.

The drying method also played a critical role in the SQ extraction. As shown in Figure 1b, hot air-dried Pandan leaves consistently yielded higher SQ content compared to freeze-dried samples. Similar results seen in research were reported that SQ content extracted under heat treatment was high [28,29]. It was also reported that heat treatment at such temperature had a minor impact on the chemical composition and quality of the extracts [30]. The increased yield is likely due to the greater disruption of cells during hot air drying, leading to easier release and more effective dissolution of targeted compounds in the extraction solvents [31]. Structural analysis of the powder from hot air-dried and freeze-dried samples before and after extraction was conducted using SEM (Figure 2a,d). Obvious differences were presented between the structures of hot air-dried and freeze-dried powders before extraction (Figure 2a,b). The hot air-dried Pandan powder (Figure 2a) exhibited more fragmented and rigid structures, in contrast to the smooth and intact structure of the freeze-dried powder (Figure 2b). These structural changes result from the destruction of fiber bundles and cellulose molecules during hot air drying, which also embrittles the material due to increased temperature [32]. 

Post-extraction SEM images (Figure 2c,d) show that UA caused considerable damage to the cell walls of samples from both drying methods, creating rough and coarse surfaces. However, powder from hot air drying exhibited more severe disruptions after UA (Figure 2c), while powder from freeze drying showed smaller cracks and damaged structures after the extraction with EH (Figure 2d). This suggests that hot air drying caused greater damage to cell walls, allowing better solvent penetration and SQ solubilization during UA, contributing to higher SQ yield.

SQ content in Pandan leaf extracts is noteworthy when compared to other plant sources. Olive oil (1.70–6.50 mg/g) extracted with supercritical carbon dioxide (over 60.00 mg/g) is also abundant in SQ [19,33]. Soybean oil and peanuts, meanwhile have lower SQ contents, ranging from 0.012 to 1.80 mg/g and approximately 0.098 mg/g, respectively [6]. Given the increasing global demand for plant-derived SQ as an alternative to marine sources, Pandan emerges as a viable and sustainable candidate. Its high SQ content, coupled with the efficiency of UA extraction, underscores its potential to meet the growing global market needs.

### 3.2. Effects of Different Drying Methods and Solvents on the Content of 2-AP

As illustrated in Figure 1d, among individual solvent systems including EtOH, IPA, and Hex, EtOH extract had the highest 2-AP content regardless of whether Pandan leaves were hot air-dried or freeze-dried. This indicated that EtOH is the optimal individual solvent for extracting 2-AP from dried Pandan leaves, a finding consistent with Azhar [34]. Additionally, the observed contents exceeded that (2.77 μg/g), reported by Laohakunjit [35], who utilized EtOH to extract 2-AP from fresh Pandan. For binary solvent systems, the highest content of 2-AP was achieved using EH, followed by EI and IH. The difference can be attributed to the significant impact of intermediate solvent polarity on extraction content, particularly evident in the binary extraction system of EtOH and Hex, which enhanced extraction performance [36]. In the case of hot air-dried Pandan leaves, the 2-AP content in the EH extract surpassed that in the EtOH extract.

Figure 1d also indicated that freeze drying led to a higher yield of 2-AP from Pandan leaves regardless of the extraction solvent used. It is likely because freeze drying preserves the papillae structure formed by the rapid crystallization of water. This finding is consistent with a study that indicated that hot air drying significantly reduces the volatile compound content in grape skins, compared to freeze drying [37]. 

However, considering that this study primarily focuses on the content of SQ from Pandan leaves, as analyzed in 3.1, hot air drying was selected over freeze drying to prepare sample for the extraction of SQ. To simultaneously obtain high content of both SQ and 2-AP, hot air drying and the extraction solvent of EH were selected for subsequent experiments.

### 3.3. Effects of the Extraction Conditions on the Content of SQ and 2-AP

#### 3.3.1. Ultrasound Time

**Figure 3:**
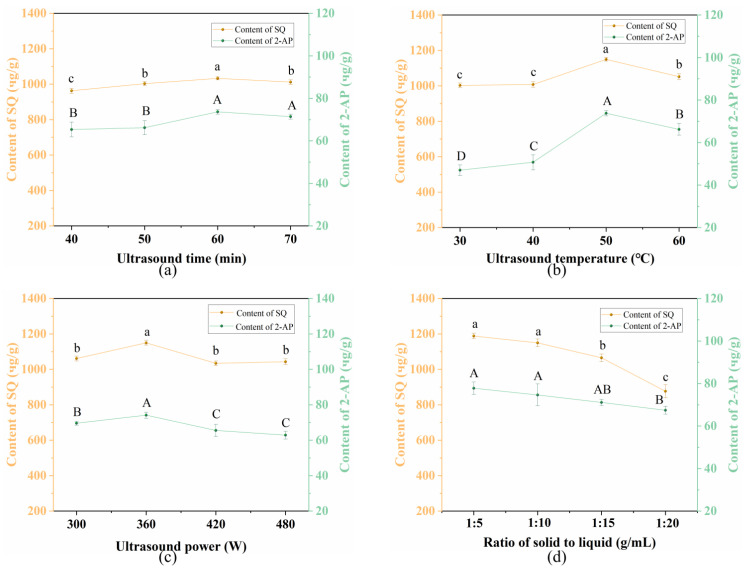
Effects of (**a**) ultrasound time, (**b**) ultrasound temperature, (**c**) ultrasound power, and (**d**) solid-to-liquid ratio on the extraction content of SQ and 2-AP from Pandan leaf extracts. Different letters on the same line mean statistically different (*p* < 0.05).

The effect of ultrasound time ranging from 40 to 60 min was examined. As illustrated in Figure 3a, both the content of SQ and 2-AP exhibited a moderate increase with an increasing ultrasound time from 40 to 60 min. However, further prolonging ultrasound time resulted in a decrease in content. This can be attributed to two factors: (1) the accumulated thermal effect accelerated the release of SQ and 2-AP from Pandan leaves leading to the increased content; (2) the longer extraction time led to higher solution concentration, resulting in reduced cellular osmotic pressure. Consequently, degradation of SQ and 2-AP in the solution occurred due to the cumulative effect of ultrasound time, leading to decreased content of SQ and 2-AP [38]. Thus, an ultrasound time of 60 min was selected for the subsequent RSM assay.

#### 3.3.2. Ultrasound Temperature

Ultrasound temperature is a critical factor influencing the target content during the UA processing. As presented in Figure 3b, the contents of both SQ and 2-AP increased significantly with the rising temperature and peaked at 50 °C (1149.87 ± 10.39 μg/g and 73.78 ± 1.24 μg/g, respectively). While further elevation of the ultrasound temperature to 60 °C led to reduced contents of SQ and 2-AP (*p* < 0.05). At higher temperatures, the cell walls of Pandan leaves became more susceptible to breakage by ultrasound cavitation, facilitating the dissolution of SQ and 2-AP and accelerating the diffusion of cell contents [39]. However, excessively high ultrasound temperatures can lead to structural degradation of SQ and volatilization of 2-AP [40], thereby lowering their contents (1051.48 ± 16.02 μg/g and 66.191 ± 2.78 μg/g, respectively). Therefore, an ultrasound temperature of 50 °C was selected as the optimal.

#### 3.3.3. Ultrasound Power

The effect of ultrasound power (300–420 W) on the extraction contents of SQ and 2-AP was also investigated. As shown in Figure 2c, both contents increased steadily from 300 W and peaked at 360 W. Afterward, the contents leveled off from 360 W to 480 W. The observed increase in contents is likely due to the generation of a vast number of cavitation bubbles at higher power levels, which induce high shear forces and microjets, thereby disrupting cells and enhancing mass transfer [41]. However, beyond the optimal ultrasound power level, both contents declined since SQ and 2-AP can be thermally degraded [42]. Consequently, the ultrasound power of 360 W was selected for further study.

#### 3.3.4. Solid-to-Liquid Ratio

The influence of solid-to-liquid ratio (1:5–1:20 g/mL) on the extraction contents of SQ and 2-AP was investigated with other conditions unchanged. Figure 3d showed that the highest contents of both target compounds were achieved at a solid-to-liquid ratio of 1:5 g/mL. SQ content decreased from 1188.26 μg/g to 876.87 μg/g with the increasing of solid-to-liquid ratio. A similar trend was observed in 2-AP content. Notably, further increasing solid-to-liquid ratio had no significant influence on the content of 2-AP using UA. While increasing to 1:15 g/mL, the content of SQ significantly decreased (*p* < 0.05). This occurred because as solid-to-liquid ratio increased, the diffusion distance of SQ from the intra-tissue increased, leading to a significant decrease in the SQ content [43]. To minimize solvent usage and waste generation, 1:5 g/mL (solid-to-liquid ratio) was selected to extract SQ and 2-AP from Pandan leaves.

### 3.4. Optimization of the Extraction Contents of SQ and 2-AP by RSM

#### 3.4.1. RSM Model Analysis

BBD was employed to determine the optimal extraction conditions focusing on three critical parameters: ultrasound time, temperature, and power. A total of seventeen batch experiments were conducted, including five central repeated experiments to assess error analysis. Experimental data were analyzed using multiple regression and the second-order polynomial equations for SQ and 2-AP content, expressed as follows:

E (μg/g) = 1219.08 + 53.24 A + 33.21 B − 39.73 C − 103.68 AB + 50.78 AC + 57.46 BC − 112.73 A^2^ − 171.77 B^2^ − 9.68 C^2^ (3)

F (μg/g) = 80.72 + 1.93 A + 2.11 B − 0.6264 C − 1.75 AB + 0.6425 AC − 1.93 BC − 2.67 A^2^ − 5.80 B^2^ − 2.05 C^2^ (4)

where E represents the content of SQ and F represents the content of 2-AP; A, B, C are the code values for the ultrasound time, ultrasound temperature, and ultrasound power, respectively.

The reliability of the second-order polynomial models was confirmed by the ANOVA results. In both models, the ρ-values (<0.0001 and <0.0008) were both <0.05, indicating the models’ strong statistical significance. Additionally, the Lack of Fit F-values were both >0.05, suggesting minimal differences between the models and the experiments. The coefficient of determination (R^2^ values) was 0.9741 and 0.9516, respectively, both close to 1. The adjusted R^2^ were 0.9407 and 0.8895, respectively, indicating the models’ strong predictive accuracy. Furthermore, the coefficients of variation (C.V. %) were 3.13 and 1.96, underscoring the precision and reliability of the experiment. In conclusion, both models were successfully established and demonstrated statistical significance and predictive reliability.

#### 3.4.2. Confirmation and Verification of Optimal Conditions

**Figure 4:**
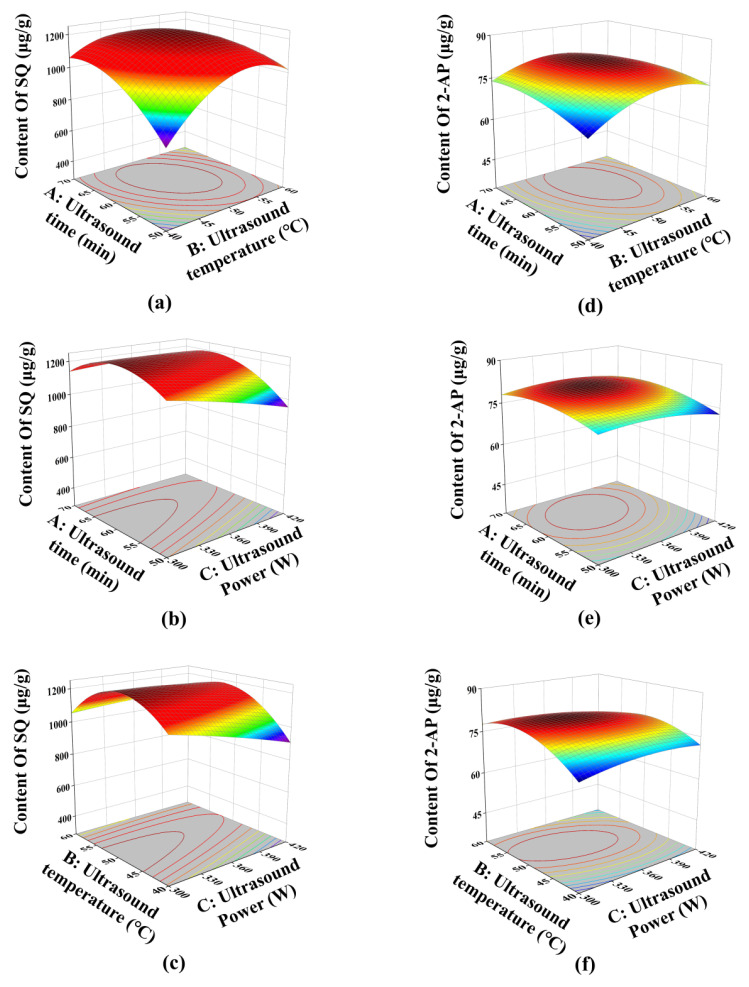
Three-dimensional response surface: (**a**) and (**d**) the interaction between ultrasound time and temperature for the content of SQ and 2-AP, respectively; (**b**) and (**e**) the interaction between ultrasound time and power for the content of SQ and 2-AP, respectively; (**c**) and (**f**) the interaction between ultrasound power and temperature for the content of SQ and 2-AP, respectively.

The interactions between two variables and their effects on the responses were visualized using the three-dimensional response surface (Figure 4). The steeper the slope of the response surface, the greater the influence of the interaction between two factors on the responses. For the response of SQ content, Figure 4a shows the steepest slope, indicating a significant influence of the interaction between ultrasound time and temperature. Figure 4b,c exhibit similar slope, illustrating comparable influences of the interactions between ultrasound time and power, and ultrasound temperature and power on SQ content.

Regarding the 2-AP content, Figure 4f displays the steepest slope, indicating that the interaction between ultrasound temperature and power had the most significant impact. In contrast, Figure 4e has a relatively gentle slope, suggesting that the interaction between ultrasound time and power had minimal effect on the 2-AP content. These results were consistent with the ANOVA analysis.

After response surface analysis, the optimal extraction conditions were: ultrasound time, 61.93 min, ultrasound temperature, 50.98 °C, and ultrasound power, 341.25 W. For practical purposes, these conditions were adjusted to ultrasound time, 62 min, ultrasound temperature, 51 °C, and ultrasound power, 300 W. Triplicate validation tests were conducted using the adjusted conditions. The content of SQ was 1229.98 ± 13.09 μg/g with a deviation of 0.12% from the model prediction. The content of 2-AP was 80.72 ± 0.88 μg/g with a deviation of 0.50% from the model prediction. These values suggest that the process of extracting SQ and 2-AP is highly reproducible and operable.

### 3.5. TPC, TFC, and TTC in the EH Pandan Leaf Extracts

**Figure 5:**
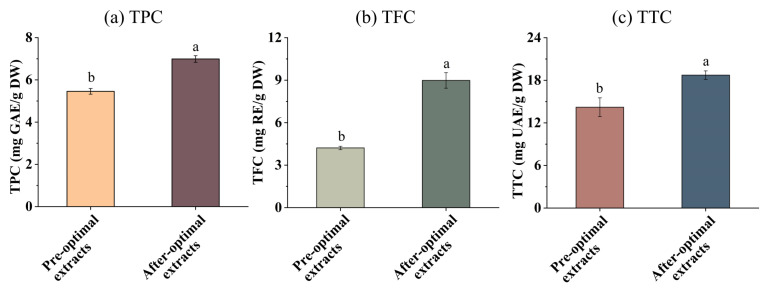
(**a**) the total phenolic content (TPC), (**b**) the total flavonoid content (TFC), and (**c**) the total Triterpenes content (TTC) in EH Pandan leaf extract before and after optimization. Different letters on the same figure mean statistically different (*p* < 0.05).

The effect of optimization on TPC, TFC, and TTC in EH Pandan leaf extracts was analyzed. As presented in Figure 5a–c, the TPC, TFC, and TTC of EH Pandan leaf extracts significantly increased after optimization compared to before optimization. This aligns with the research of Zampar [44], who described that UA conditions significantly influence TPC, which ranged from 326.76 to 415.06 mg GAE 100/g with the ultrasound time from 5 to 30 min. A similar trend was also seen in TFC. Additionally, TTC increased with the SQ content after optimization, as SQ is a natural triterpene.

### 3.6. Antioxidant Activities of the Pandan Leaf Extract

At 517 nm, the highly stable free radical DPPH shows a strong absorption peak in the ethanol solution. When mixed with antioxidants, the color of the solutions lightens, indicating the scavenging ability of the antioxidant. This method is widely used for its high sensitivity and efficiency. Similarly, the ABTS scavenging activity assay is widely used to determine antioxidant activity in samples for its simplicity and rapidity [45,46]. Metal ions can catalyze oxidation; thus, the reducing power of ferric iron and cupric ions was measured to assess the reducing power. Due to the antioxidant properties of SQ, there is a significant market demand for it. Thus, the antioxidant activity of EH Pandan leaf extracts enriched in SQ, both before and after optimization, were comprehensively evaluated using various assays (DPPH, ABTS, FRAP, and CUPRAC assay).

**Figure 6:**
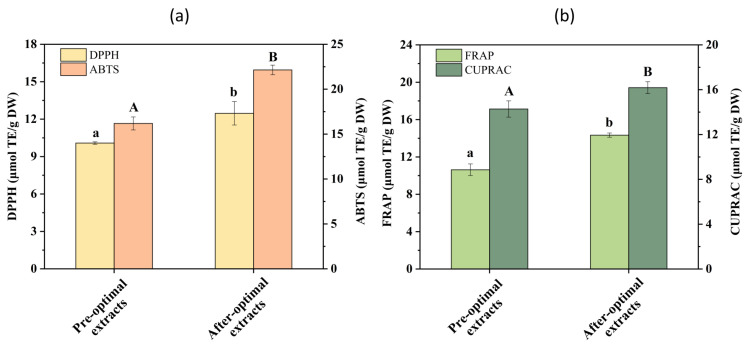
Antioxidant activities in the Pandan leaf extracts of pre-optimization and after optimization determined using DPPH, ABTS radical scavenging assays (**a**), FRAP, and CUPRAC assays (**b**). Different letters on the same color figure mean statistically different (*p* < 0.05).

All assays showed a similar trend. As shown in Figure 6a, the values from the DPPH and ABTS assay of the post-optimization extracts were 12.46 μmol TE/g DW and 22.14 μmol TE/g DW, respectively. With comparison to pre-optimization, this is an increase of 23.66% and 37.83%, respectively. These results suggest that the compounds in EH extracts from Pandan leaves can donate electron or hydrogen atoms to scavenge DPPH radicals [47] and the compounds contribute to the ABTS scavenging activity. This is likely due to SQ which has a significant influence on these processes, as in vitro and in vivo studies have highlighted the antioxidant effect of SQ owing to its molecular properties [48]. To investigate the reducing power, FRAP and CUPRAC assays were performed. As shown in Figure 6b, the reducing power of the post-optimization extracts (FRAP: 14.275 μmol TE/g DW, CUPRAC: 16.181 μmol TE/g DW) was greater than that of the pre-optimization extracts (FRAP: 10.629 μmol TE/g DW, CUPRAC: 14.275 μmol TE/g DW). SQ as a natural triterpene has been shown to correlate positively and significantly with various antioxidant assays. It has been reported that SQ-rich Sacha inchi (*Plukenetia volubilis*) seed oil exhibited antioxidant activities, with DPPH and ABTS assays yielding values of 3.23 μmol TE/g oil and 2.10 μmol TE/g oil [49]. The extracts of Pandan leaves in the current study exhibited higher values than those of Sacha inchi seed oil, meaning that SQ-rich Pandan leaf extract may be an efficient antioxidant.

### 3.7. Hypoglycemic Activity of the Pandan Leaf Extract

**Figure 7:**
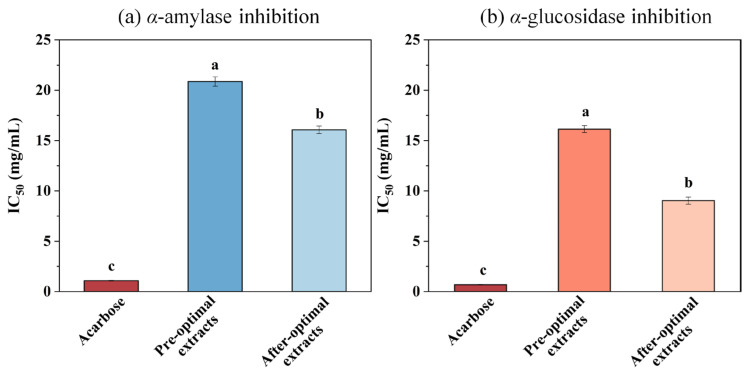
(**a**) *α*-amylase and (**b**) *α*-glucosidase inhibition (IC_50_ values in mg/mL, data were obtained from triplicate experiments and expressed as mean ± STD) of acarbose and Pandan leaf extracts before optimization and after optimization. Different letters on the same figure mean statistically different (*p* < 0.05).

The *α*-amylase and α-glucosidase inhibition activities were measured to EH Pandan leaf extracts. The results were presented as the IC_50_ values with acarbose serving as the positive control (Figure 7). Figure 7a illustrates that the IC_50_ value of the pre-optimization extract was significantly higher than the IC_50_ value of the post-optimization extract, and both were significantly higher than that of acarbose. Figure 7b illustrates the inhibitory activity of *α*-glucosidase. The IC_50_ values of the Pandan leaf extract were higher than that of acarbose, but the post-optimization Pandan leaf extract had a lower IC_50_ value than the pre-optimization extract, indicating that the SQ-rich Pandan leaf extract has a good inhibitory effect on *α*-glucosidase. These results are similar to the study of Conforti [50], who reported that n-hexane extract of squalene-rich *amaranth* seeds effectively inhibited the *α*-amylase activity. In addition, Sanni used molecular docking and demonstrated that SQ from *Azadirachta indica* has an inhibitory effect against *α*-amylase and *α*-glucosidase enzymes [51].

Pancreatic *α*-amylase catalyzes the hydrolysis of the *α*-1, 4-glycosidic bond, thereby initiating the catalysis reaction of the starch hydrolysis, which plays an important role in the digestive system [52]. While *α*-glucosidase further breaks down the disaccharides into monosaccharides which are readily absorbed in the intestine. Thus, it is considered a therapeutic target for the regulation of postprandial hyperglycemia [53], an early metabolic abnormality in type 2 diabetes. Widyawati [54] investigated the hypoglycemic effect of SQ extracted from *Syzygium polyanthum* leaf in a rat model of streptozotocin (STZ)-induced diabetes. 

Acarbose, a currently commonly used drug for treating diabetes, can cause side effects such as bloating due to its strong inhibitory activity on starch-hydrolyzing enzymes, leading to the accumulation of undigested carbohydrates [55]. Therefore, finding a new *α*-amylase and α-glucosidase inhibitor with moderate inhibitory effects and fewer side effects than acarbose would be advantageous. Our results suggest that the SQ-rich Pandan leaf extract can help reduce postprandial hypoglycemia using intro assays, making Pandan a potential plant-based alternative for preventing and treating hypoglycemia.

## 4. Conclusions

This study highlights the effectiveness of different drying methods and extraction systems for maximizing the yields of SQ and 2-AP from Pandan leaves. In summary, hot air drying was found to be more effective for extracting SQ, while freeze drying was better suited for preserving 2-AP. For hot air-dried Pandan leaves, the binary solvent system of EH was identified as optimal for the simultaneous extraction of both SQ and 2-AP using UA. Under the optimal UA conditions (ultrasound time of 60 min, temperature of 50 °C, power of 300 W, and solid-to-liquid ratio of 1:5 g/mL), the contents of SQ and 2-AP reached their highest levels at 1229.984 ± 13.092 μg/g and 80.718 ± 0.884 μg/g, respectively. Additionally, Pandan leaf extracts exhibited strong antioxidant activities, as evidenced by significant free radical scavenging activity against DPPH and ABTS. The extracts also demonstrated a substantial reduction in the power for ferric and cupric ions and effectively inhibited the enzymatic activities of *α*-glucosidase and *α*-amylase. This study provides valuable information and insights into extracting bioactive compounds from Pandan leaves, supporting their potential use as natural sources of antioxidants and functional food ingredients. The optimized extraction process enhances the economic and therapeutic value of Pandan, paving the way for its broader application in health and nutrition industries as an antioxidant and hypoglycemic agent. Future studies shall consider in vivo experiments to validate the antioxidant and hypoglycemic effects of Pandan extracts.

## Figures and Tables

**Table 1:** Coded and uncoded levels of the three independent variables.

Factor Level	Independent Variables		
	Extraction Time (A/time)	Extraction Temperature (B/°C)	Extraction Power (C/W)
−1	50	40	300
0	60	50	360
1	70	60	420

## Data Availability

The original contributions presented in this study are included in the article. Further inquiries can be directed to the corresponding author.
